# Positive Feedback Promotes Oscillations in Negative Feedback Loops

**DOI:** 10.1371/journal.pone.0104761

**Published:** 2014-08-15

**Authors:** Bharath Ananthasubramaniam, Hanspeter Herzel

**Affiliations:** Institute for Theoretical Biology, Charité and Humboldt-Universität zu Berlin, Berlin, Germany; Tata Institute of Fundamental Research, India

## Abstract

A simple three-component negative feedback loop is a recurring motif in biochemical oscillators. This motif oscillates as it has the three necessary ingredients for oscillations: a three-step delay, negative feedback, and nonlinearity in the loop. However, to oscillate, this motif under the common Goodwin formulation requires a high degree of cooperativity (a measure of nonlinearity) in the feedback that is biologically “unlikely.” Moreover, this recurring negative feedback motif is commonly observed augmented by positive feedback interactions. Here we show that these positive feedback interactions promote oscillation at lower degrees of cooperativity, and we can thus unify several common kinetic mechanisms that facilitate oscillations, such as self-activation and Michaelis-Menten degradation. The positive feedback loops are most beneficial when acting on the shortest lived component, where they function by balancing the lifetimes of the different components. The benefits of multiple positive feedback interactions are cumulative for a majority of situations considered, when benefits are measured by the reduction in the cooperativity required to oscillate. These positive feedback motifs also allow oscillations with longer periods than that determined by the lifetimes of the components alone. We can therefore conjecture that these positive feedback loops have evolved to facilitate oscillations at lower, kinetically achievable, degrees of cooperativity. Finally, we discuss the implications of our conclusions on the mammalian molecular clock, a system modeled extensively based on the three-component negative feedback loop.

## Introduction

**Figure 1:**
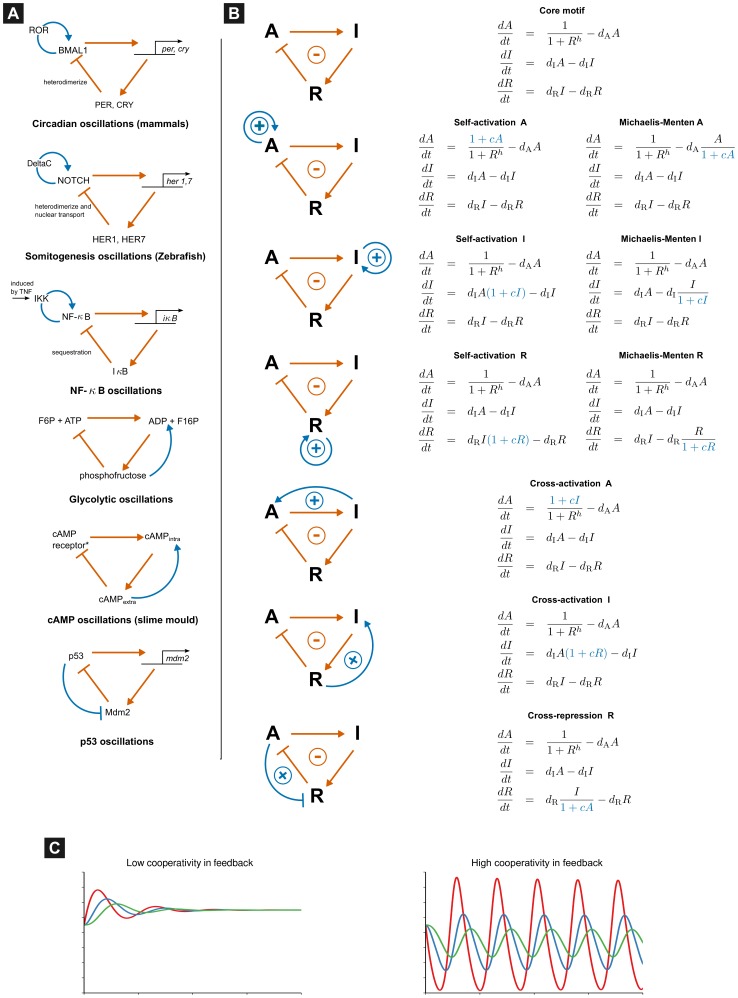
Instances of three-component negative feedback loops with positive feedback in biochemical oscillators and the generic motifs studied in this work. (A) Some biochemical oscillators utilizing the three-component negative feedback loop motif. Positive feedback loops (in blue) are often found along with the core negative feedback loop (in orange) (B) Oscillatory motifs involving the three-component negative feedback loop (*top*) with all possible auxiliary positive feedback loops (in blue). The motifs encountered in biochemical oscillators are clearly recognizable. The kinetic model formulation for each motif is also listed with the term representing the positive feedback in blue. The three components of the negative feedback loop are activator 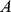, intermediate 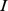 and repressor 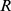. The parameter 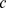 tunes the strength of the positive feedback, with a value of 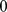 representing no positive feedback. (C) Waveform of the Goodwin oscillator with equal degradation in all steps with cooperativity increased from 3 (*left*) to 10 (*right*).

The identification of motifs within biological networks and assignment of function to those motifs has been a key undertaking of Systems Biology [1], [2]. One of the classic motifs capable of sustained oscillations using negative feedback interactions alone is the three-component loop [3]–[6] (in the Analysis section, we describe a systematic approach to identify such positive and negative feedback loops in a system). Several prominent biochemical oscillations have been attributed to this motif including those in eukaryotic circadian systems [7], somitogenesis [8], glycolysis [9], cAMP signaling [10], DNA damage response (p53) [11], cellular stress response (NF-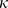
B) [12], [13] and the synthetic repressilator (see Figure 1A). The glycolytic [14], [15] and cAMP oscillators have been alternatively attributed to a two-component negative feedback motif (the choice of motif length depends on the components and processes considered essential in the model). Interestingly, the core negative feedback motif is augmented with positive feedback loops in several of these systems [3]. This raises the question of the evolutionary purpose of these auxiliary positive feedback loops. Tsai et al. [16] have suggested that positive feedback loops provide robustness and tunability to oscillations in the system without elaborating on the kinetic mechanism by which such positive feedbacks are beneficial. In this work, we address the principles by which these positive feedback loops are beneficial in biochemical oscillations and unify several common mechanisms known to promote oscillations, such as Michaelis-Menten [17] degradation kinetics [18] and self-activation.

We use a minimal well-studied formulation of this three-component negative-feedback loop motif called the ‘Goodwin oscillator’ [5] for this theoretical study. The delay provided by three biochemical steps and cooperativity in the negative-feedback are essential for oscillations. This cooperativity can result from cooperative binding [19], [20], allostery [21], reversible covalent modification [22] or sequestration [23], [24]. Cooperativity is also intimately related to ‘ultrasensitivity’ and ‘nonlinearity’ in the biological modeling literature. The degree of cooperativity in the feedback is measured by the Hill coefficient; the larger the value of the coefficient, the higher the degree of cooperativity.

One long-recognized limitation of this negative feedback only motif is the need for a high degree of cooperativity (a Hill coefficient of at least 8) to produce oscillations [6]. The complexity involved in evolving such high degrees of cooperativity is unknown and experimentally-measured Hill coefficients are in the range 2–4 [25]. Nevertheless, mechanisms have been suggested that are theoretically capable of producing effectively high degrees of cooperativity, such as covalent modification (multi-site phosphorylation) [22], [ 26] and sequestration [27]. Extending the length of the loop with additional steps is another way to alleviate the high degree of cooperativity required. In this work, we show that positive feedback loops provide a powerful alternative way of reducing these requirements, irrespective of the mechanism effecting this positive feedback. We do this by keeping stringent accounts of the nonlinearities in the core three-component motif and identifying cooperativity as the key metric to compare different positive feedbacks. We conjecture that this facilitation of oscillations explains the frequent occurrence of positive feedbacks within these negative feedback systems.

## Results

We consider the three-component negative feedback loop motif represented by the Goodwin oscillator with various auxiliary loops listed in Figure 1B. This motif is simple enough to allow theoretical analyses of its oscillatory properties. Nevertheless, properties of the motifs for different choices of kinetic parameters require numerical evaluations of analytically-derived conditions. We call the three components in the motif, the activator (A), intermediate (I) and feedback repressor (R). The positive feedbacks can be grouped into three classes based on the underlying mechanism: self-activation (SA), Michaelis-Menten (MM) degradation and cross-activation (CA). Here, we consider the simplest possible mathematical representations of the motifs (shown in the right column of Figure 1B) to emphasize the generality of our result. However, more complex interactions in systems with Jacobians having patterns of nonzero elements described in the Analysis section will also obey our observations. We empirically test the properties of 2000 kinetic rate parameter choices (chosen randomly using latin hypercube sampling to explore well the space of parameters) for each motif in Figure 1B. A similar Monte-Carlo approach was used to study the conditions for oscillations in arbitrary metabolic networks in [28]. In order to obtain general conclusions, we compare different motifs holding common kinetic parameters at identical values. Since we use non-dimensionalized models, all metrics presented in this Results section are without units.

### Positive feedback motifs require smaller degree of cooperativity to oscillate

**Figure 2:**
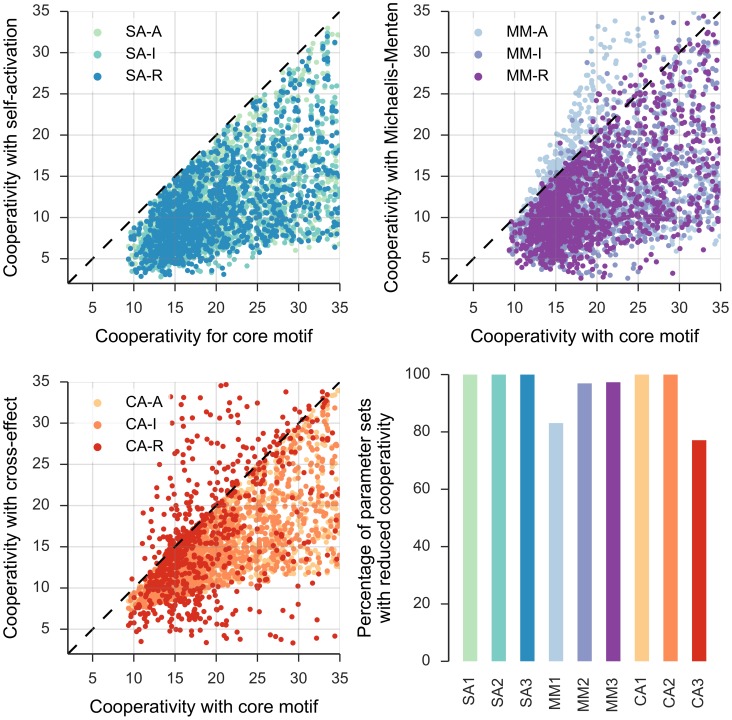
Minimum cooperativity required for oscillations for the core motif versus the positive feedback motifs. The motifs with positive feedback are grouped according to the type of mechanism: self-activation (SA), Michaelis-Menten (MM) degradation or cross-activation (CA). The component (activator, intermediate or repressor) on which the positive feedback is acting is also indicated by color. Each point represents one choice of parameters for the motif and the comparison is made with common parameters having identical values. In the scatter plots, points below the y = x line represent cases where the positive feedback motif oscillates at a smaller cooperativity than the core motif. The data in the three scatter plots is summarized in the bar graph, i.e., the percentage of parameter sets where positive feedback motifs oscillate with smaller cooperativity than the core motif.

We first study the minimum degree of cooperativity required to produce oscillations in all motifs in Figure 1B. The degree of cooperativity of a motif is measured by the Hill coefficient in the negative feedback regulation. For each choice of parameters, the positive feedback motifs are compared against the core motif without positive feedback (Figure 2). Generally, addition of any positive feedback loop reduces the required cooperativity to produce oscillations. Among the classes of motifs considered, SA motifs always oscillate with lesser cooperativity than the core motif. On the other hand, MM degradation and CA motifs oscillate with smaller cooperativity for most, but not all considered parameter choices. The amount of improvement in the cooperativity required also depends upon the motif (see Figure S1).

For some parameter choices, positive feedback loops are not beneficial but rather detrimental, i.e., they increase the cooperativity requirement. In MM motifs, the detrimental parameter choices correspond to positive feedback on the most stable (longest lifetime) component. In this situation, the effective lifetime of the most stable component is lengthened further leading to increased mismatch between the lifetimes of the three components. This mismatch between component lifetimes is known to drastically increase the degree of cooperativity needed for oscillations even in the core motif [6], [ 29].

The effect of positive feedback can also be visualized using bifurcation diagrams to verify that the cooperativity required decreases continuously with increasing positive feedback strength (Figure S2). We use the parameter *c* in motifs (see Figure 1B) as a measure of the positive feedback strength. In all the SA motifs, CA-A and CA-I motifs, stronger feedback leads to smaller required cooperativity consistent with earlier observations. However, for the MM and CA-R motifs, weak positive feedback has a beneficial effect, while stronger positive feedback is detrimental. This suggests that points above the diagonal (i.e., with higher cooperativity with positive feedback) in Figure 2 represent parameter choices corresponding to strong positive feedback.

The positive feedback strength *c* in the MM class of motifs allows comparison between systems with zero-order, first-order, and intermediate-order degradation. First-order degradation corresponds to *c* = 0, intermediate-order of degradation *c*>0 and zero-order degradation 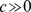. As seen in Figure S2, going from first-order to intermediate-orders of degradation makes oscillations achievable at lower cooperativity in the MM class of motifs. However, zero-order degradation is not as beneficial as intermediate-order MM degradation in producing oscillations. In other words, there is an optimal order of degradation for each choice of parameters, where oscillations are possible at the lowest degree of cooperativity (evident when Figure S2 is shown for larger range of *c*).

These observations can also be derived by the theoretical analyses presented in the Analysis section.

### Short half-life components are prime candidates for beneficial positive feedback

We showed in the previous section that the addition of positive feedback loops reduces the burden of nonlinearities in the system. We next evaluate which positive feedback produces the largest reduction in the cooperative requirement within a class of motifs for a given choice of parameters. In other words, for which positive feedback is the largest payoff obtained?

**Figure 3:**
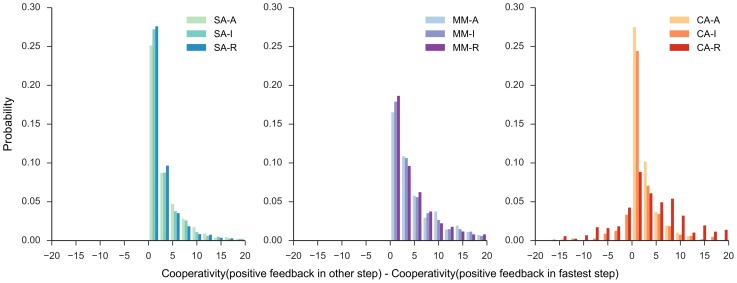
Reduction in the required cooperativity among positive feedbacks within each class. For each class, the additional reduction achieved in the minimum cooperativity by placing the positive feedback on the fastest step (largest degradation rate or smallest half-life) in the three-component motif, relative to positive feedback elsewhere, is shown as a histogram for the same data in Figure 2. For the class CA, this translates to a positive feedback between the two fastest steps in the core motif. For each choice of parameter values, the motif that happens to have the positive feedback on the fastest step (or between the fastest steps) is compared against the remaining two motifs within each class and labeled with name of the former. We compare different motifs keeping common parameters at identical values using the color scheme used in Figure 2.

For all three classes of motifs considered, positive feedback on the fastest dynamic (or shortest half-life) component in the three-component core motif produces the largest reduction in the cooperativity requirement (Figure 3); for the CA motifs, the most favorable feedback is between the fastest two dynamic components (out of the three). For the SA and MM motifs, the mismatch between component degradation rates (or half-lives) is the primary driver of high cooperativity (the mismatch is found on the right hand side of (7) and (9)). The positive feedback on the fastest component reduces that component's effective degradation rate and thus reduces the mismatch between the three components. Moreover, for the CA motifs, the mismatch cost on the cooperativity requirement (right hand side of (12)) is reduced in proportion to the degradation rate of the fastest two steps and hence, a cross activation between the fastest two steps is favored.

The CA-R motif, in up to 39% of the cases, had a smaller cooperativity requirement than a motif with positive feedback between the fastest two steps. In the CA-R motif, since the positive and negative feedback act between the same components, the reduction of the mismatch cost is confounded by the degree of cooperativity (see (12)). Therefore, there is no clear trend in the benefit of CA-R motif across different lifetimes of the components.

### Oscillations with periods longer than dictated by component half-lives possible

**Figure 4:**
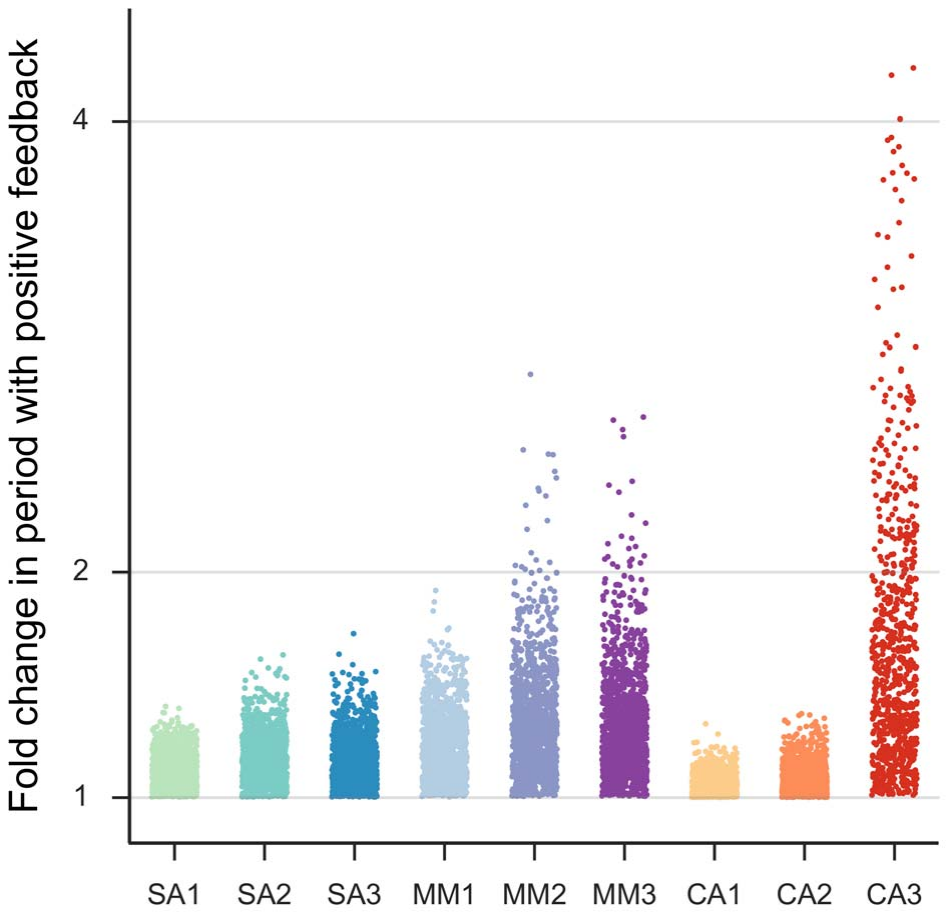
Period of oscillations for the core motif versus the positive feedback motifs. The ratio of the period of the positive feedback motif and period of the core motif for different choices of parameter values categorized by class: SA, MM and CA.

The period of oscillations of the core motif is dependent only on the lifetimes of the three components in the loop. Thus, biological oscillators with the core motif can oscillate only with periods in the same order of magnitude as the component degradation rates. However, autonomous circadian oscillators have a period of 24h, despite constituent mRNA and protein half-lives being in the order of at most few hours. The positive feedback motifs, we consider, have consistently longer periods than the core motif for the same choice of parameters (see Figure 4). In other words, periods of oscillation longer than that expected based on component half-lives alone are possible, such as circadian and infradian rhythms.

### The effects of multiple positive feedbacks are cumulative

**Figure 5:**
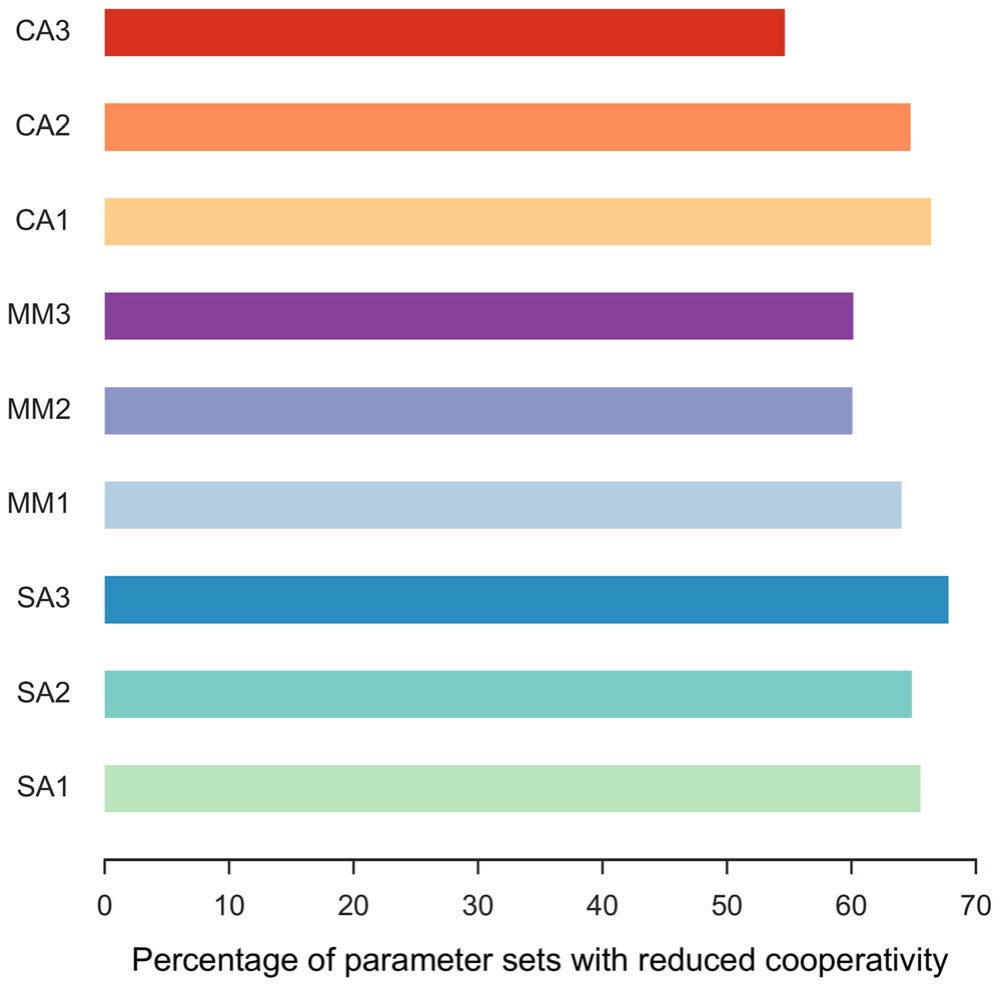
Cumulative effect of two positive feedback loops on the core motif. The percentage of random parameter choices for which the cooperativity was improved by adding a second positive feedback to the nine positive feedback motifs in Figure 1. The original positive feedback motifs (shown on the y-axis) were modified by adding a second positive feedback different from the first, i.e., each of the other eight.

Our results indicate that positive feedback loops reduce the cooperativity requirement with a likelihood and extent that is dependent on the particular motif and its parameterization. In many instances of biological oscillators, multiple feedback loops are observed in combination. We tested whether combinations of motifs in Figure 1B perform better than the nine basic positive feedback motifs. We compare each of the nine basic motifs with and without one of the other eight positive feedback motifs. The percentage of random parameter choices that yielded a further decrease in the required degree of cooperativity are shown in Figure 5. For a fairly large fraction (on average about 60%) of random parameter choices the improvements (i.e., reduction in cooperativity) were cumulative. Therefore, if the parameters were further chosen appropriately, the cooperativity can be reduced systematically using multiple positive feedback motifs.

## Discussion

In this paper, we illustrated the ability of three classes of positive feedback loops to facilitate oscillations in a generic delayed negative feedback motif. We were primarily motivated by the dual observations that several oscillatory negative feedback motifs are accompanied by positive feedback loops, and models of simple oscillators require high degrees of cooperativity rarely observed in experiments. We showed in the classic three-component Goodwin core motif [5] that the addition of positive feedback loops reduces the nonlinearity required in the negative feedback as measured by the degree of cooperativity. The positive feedbacks facilitating oscillations result from common kinetic mechanisms, such as self-activation and enzymatic MM degradation. Moreover, the benefits of multiple positive feedbacks are additive in a majority of the cases. The period of oscillations in the positive feedback motifs is always larger than the underlying core motif.

Tsai et al. [16] suggested that the addition of positive feedback loops provide robust oscillations of constant amplitude tunable over a wide range of periods using several oscillator models. This result holds for oscillators operating under the relaxation regime with highly non-sinusoidal waveforms, very different from the regime we study, and they leave open the question of the role of positive feedback on cooperativity in the negative feedback. In particular, their theory is unable to explain situations where robust non-tunable periods are necessary, such as the circadian oscillator, and the possible kinetic mechanism by which positive feedback facilitates oscillations.

Comparing the robustness of ten different oscillator models, Wolf et al. [30] concluded that negative feedback-based oscillators (for e.g., the circadian models they consider) are more robust. They further suggest that, in Goodwin-like negative feedback loops with different numbers of intermediates, positive feedback makes the system less robust, in contradiction to Tsai et al. [16]. However, while the size of the parameter region of oscillatory behavior is used to measure robustness in [16], local period sensitivity is the robustness metric in [30], which might explain the discrepancy. Wolf et al. show, nevertheless, that lengthening the negative feedback loop can improve robustness, a mechanism that has also been shown to reduce the cooperativity requirement [31].

Kholodenko [32] showed that oscillations are possible in the MAP kinase (MAPK) signaling pathway with a negative feedback from the final product (a doubly-phosphorylated kinase) to the first kinase in the chain. In this system, MAPK phosphorylation cascade provides the cooperativity that along with the negative feedback produces oscillations as discussed throughout this paper. Interestingly, the number of levels in the phosphorylation cascade determines the degree of cooperativity within the structure. Moreover, the MM kinase/phosphatase kinetics used in this model aid in producing this ultrasensitivity much like explicit positive feedback [33].

### Molecular circadian clock

**Figure 6:**
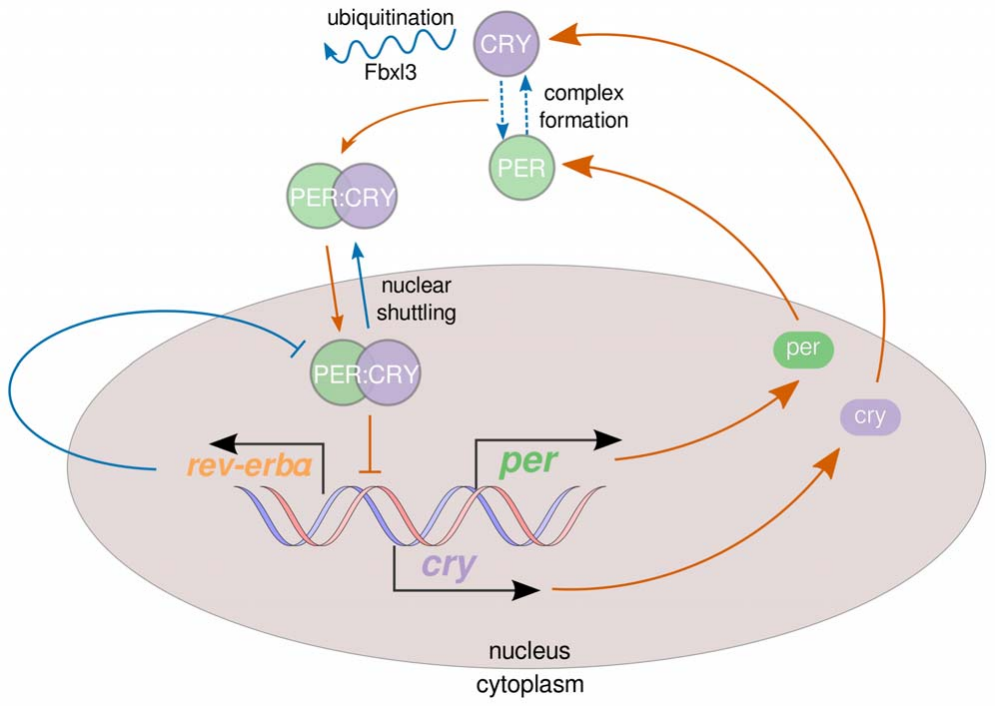
Components of the mammalian circadian oscillator. Interactions within the clock network have been reduced in order to make simple motifs from Figure 1 easy to identify. We use the same color for positive and negative interactions as in Figure 1, i.e., orange for the negative feedback loop and blue for the positive feedbacks.

We explore the implications of our insights in the context of the cellular circadian oscillator in mammals. Circadian clocks in other eukaryotes consist of similar components and interactions and thus, the following discussion is applicable to those organisms as well. The cell-autonomous circadian oscillator consists of certain ‘core-clock’ genes, *per* and *cry*, that are transcribed, translated, and finally inhibit their own transcription (see Figure 6). Delays in this feedback are due to cellular processes, such as post-translational modifications, complex formation and nuclear transport [34]. As seen in the figure, the core feedback loop resembles the core Goodwin motif and this feature was exploited as such in several early iterations of circadian oscillator models [7], [ 35], [ 36].

While this core negative feedback loop involving only *per* and *cry* is potentially capable of producing oscillations, transcriptional repression that closes the loop would need to have a very high cooperativity (at least 8) to be capable of sustained oscillations [6]. Such high cooperativity in transcriptional regulation appears unrealistic as indicated earlier, although it could be realized by cooperative binding at several binding sites or post-translational modification, such as multi-site phosphorylation [37] or sequestration [38]. Moreover, the components in this feedback loop (mRNA and proteins) have degradation rates of a few hours, which is fast relative to the timescale of the observed near 24h circadian oscillations. Fortunately, this core Goodwin-like motif is augmented with several direct or implicit positive feedback loops that we argue collaboratively alleviate this cooperativity requirement.

The protein product of the gene 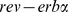 that is repressed by the PER and CRY proteins also represses *per* and *cry* transcription via the transcriptional activator BMAL1. This double negative interaction is one direct positive feedback loop in the scheme of the cross-activating motif CA-R. The degradation of the clock proteins (for instance, CRY via ubiquitination mediated by Fbxl3) is an enzymatic process and thus is likely governed by MM kinetics (motif MM2). Another feature of the molecular clock is that PER and CRY enter the nucleus and repress transcription not as monomers, but as a heterodimer. In other words, there are two parallel Goodwin-like loops that are coupled via dimerization.

Such a complex formation can be considered a positive feedback interaction as well. Looking from the perspective of CRY, increased CRY repressor reduces PER protein, which in turn up-regulates *cry* transcription; in effect a positive feedback loop. By the symmetry of the system, a similar argument holds from the perspective of PER as well.

This complex formation also adds another step in the feedback loop, which also eases the cooperativity requirement. The PER:CRY complex also shuttles across the nuclear membrane. This adds another step to the loop, since the reversible step behaves like a cross-activating positive feedback loop (CA-I) as long as there is asymmetry between the forward and backward steps.

As we have shown, the benefits of different positive feedback loops do add up to ease the cooperativity requirement. Here we have highlighted a few important positive feedback interactions in the mammalian circadian clock, but the state-of-the art network of interactions is very complex encompassing additional positive and negative feedback motifs.

Quantitative modelers of the molecular circadian oscillator have long recognized the need for ‘modifications’ to the core Goodwin motif model [7] to have more plausible cooperativity requirements. MM degradation kinetics [17] for one or more components representing proteins is the most common approach used in these models to reduce the cooperativity to at least 4 [35], [ 39]–[41]. In fact, Tyson et al. [41] reduced the cooperativity of the feedback to 2 by introducing dimerization (a form of complex formation) and Michealis-Menten-like degradation of the proteins motivated by biological observations. Some features identified as positive feedback-like mechanisms, such as nuclear transport and hetero-dimerization, were also suggested as mechanisms that promote oscillations in clock models based on the core Goodwin motif by Kurosawa et al. [42]. In fact, Kurosawa et al. compared four different model architectures also using cooperativity in the feedback as a measure of ‘ease’ of obtaining oscillations. Protein sequestration has been shown to be capable of generating highly cooperative signaling responses [24], [ 27], such as those needed for sustained oscillations with no other nonlinearity. Kim and Forger [38] showed using their model that sequestration of the repressor by the activator (analogous to the CA-R motif) enable oscillations in the molecular circadian clock by requiring a stoichiometric balance between activators and repressors in the system.

### Reducing cooperativity in the feedback

Griffith [6] first showed that cooperativity of at least 8 was necessary to produce oscillations in the core Goodwin motif. Subsequently, Tyson and Othmer [31] presented the exact relationship between the cooperativity in the negative feedback and length of the enzymatic chain (so called secant condition). Thus, they confirmed generally that cooperativity could be reduced by increasing the length of the feedback loop, i.e., adding more steps. Similarly, Bliss et al. [18] showed that the required cooperativity could be reduced by explicit time-delay in the loop and saturable end-product removal (MM-like kinetics).

From the analysis of a delayed negative feedback oscillator [43], we notice that the period of oscillations is primarily determined by the feedback delay. Thus, the longer periods observed in the positive feedback motifs can explain the smaller degree of cooperativity required for these motifs to oscillate. The positive feedback loop increases the effective length of the feedback delay allowing lesser cooperativity to suffice. This view is also consistent with our observation that positive feedback motifs increase the effective half-life of the fastest component in the three component loop.

Thron [29] reinterpreted the secant condition based on chemical reaction orders for enzymatic chains with feedback between the last and first substrates. Interestingly, Thron noted with an example that enzymatic chains without cooperativity (a Hill coefficient of 1) can oscillate with saturable substrate removal (MM-type enzymatic kinetics) and appropriate choice of parameters. Kurosawa and Iwasa [44] studied theoretically the effect of MM enzymatic kinetics on the oscillations in circadian models. They concluded, consistent with our study, that having saturating kinetics in the degradation reactions promoted oscillations, whereas having them in the activating terms makes oscillation less likely. We can rephrase their findings within our paradigm as: when MM kinetics acts like positive feedback (when in the branch degradation reactions) it aids oscillations, whereas when MM kinetics acts as negative feedback (when in the activating loop reactions), it is detrimental to oscillations.

Self-activation, or product activation as it is also known, is also encountered in several biological oscillator motifs. Goldbeter and Dupont [21] investigate the role of cooperativity generated by allosteric enzyme modifications in glycolytic and Ca^2+^ oscillations. Significantly, they identify that positive feedback generated by product-activated enzyme along with MM kinetics in the substrate removal can produce oscillations without need for cooperativity. Although the models they consider for these two systems do not need cooperativity, they include other nonlinearities, such as multiplicative terms and competitive inhibition.

The cross-activating motifs (CA-A, CA-I) have been shown to produce oscillations under the alternative name of amplified negative feedback in [4]. Marteil and Goldbeter use a combination of MM degradation of the inhibitor and cross-activation to reduce the cooperativity in the model for cAMP oscillations in slime mold to about 2.

### Alternative views on the effect of positive feedback

As is evident from our theoretical analysis and discussed earlier by Thron [29], one of the drivers of high cooperativity requirement is the mismatch between the effective degradation rates between the components, albeit measured at the critical point. Positive feedback reduces the needed cooperativity by reducing the mismatch between the component degradation rates. This is manifest as the prescription we presented that the positive feedback must be placed in the step with shortest half-life to obtain the best benefit. We might also speculate that such positive feedback on the fastest step would be (and have been) favored by evolution as they produce oscillations most easily (or, with the weakest positive feedback). Since positive feedback only has the ability to lengthen lifetimes, the mismatch can be reduced only by slowing down the fastest steps. In the case of the CA motifs, there are additional benefits of positive feedback in the form of reduction of the mismatch cost.

It is well known that nonlinearity is critical to generating oscillatory phenomena [3]. The measure of cooperativity can then be considered to be a measure of this nonlinearity required for oscillations. The positive feedback motifs in effect redistribute the ‘total’ nonlinearity across multiple steps, which is apparent under the reaction order formulation of Thron [29] for the MM and SA motifs. This distribution is further beneficial, since the effective reaction order (or cooperativity) of the system is a product rather than a sum of the individual reaction orders.

Classical models of biological pattern formation [45] work on the principle of short-range activation and long-range inhibition. Thus, spatio-temporal patterns are generated by auto-catalysis (positive feedback) and long range inhibition (negative feedback). If (periodic) oscillations are viewed as a purely temporal pattern, it is not surprising that positive feedback enhances negative feedback oscillations.

### Concluding Remarks

While we have focused here on understanding the role of positive feedback and suggesting its evolutionary purpose in easing oscillations by reducing the cooperativity requirements, our results can be also viewed in a prescriptive light. Given a model of an oscillatory biological phenomenon, unreasonably high cooperativity values within the model would suggest that one or several important positive feedback mechanisms have been overlooked in model construction. This might also direct further experimental work if evidence of such positive feedback is lacking.

It has been also suggested that positive feedback provides robustness to the system [16]. This robustness is measured using the fraction of random parameter choices within some space that result in stable oscillations. The idea being that this metric is a surrogate for the size of the parameter space where oscillations occur. The larger the parameter space with oscillations, the more robust the oscillations are to changes in parameter values. However, estimating the size of the parameter space using this approach is difficult, since (i) models oscillate in different regions of parameter space and (ii) the size of a region of parameter space is meaningful only relative to the observed range of variation of that parameter that depends on the biological process it represents. Therefore, we did not test the ‘robustness’ of the motifs in this work.

We have elaborated on the qualitative role of positive feedback in reducing the nonlinearity necessary to produce oscillations using the simple Goodwin core motif. This three-component negative feedback is a common motif in several biochemical oscillators [4] and more complex oscillators can be reduced to this three-component motif with positive feedback. We exploited the advantage of simple motifs to capture general functional roles [2] of feedback structures, without the complexity that accompanies studies of specific systems. Moreover, we implemented these motifs using plausible yet generic mathematical representations. However, checking the mathematical formulation of more complex functional relationships exhaustively is beyond the scope of this work. We did not include three-component motifs with three negative feedback interactions due to a technical difficulty. Such a motif requires a nonlinearity in each component due to the requirement that all three components always remain positive. Therefore, attributing a single metric, such as a degree of cooperativity, to the motif as a whole was not possible, as was needed to test our hypothesis. Nevertheless, our qualitative conclusions apply to a vast majority of motifs encountered in feedback oscillators.

## Analysis

The original Goodwin oscillator represents a mechanism involving a three-component negative feedback loop:
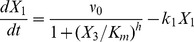


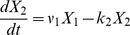


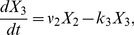
(1)where 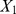,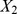, 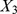 are the concentrations of mRNA, protein and end-product. All the dynamic properties of the original system (1) is captured by the rescaled version of this model having fewer parameters:
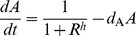


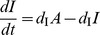


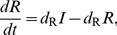
(2)where the three components are called activator 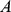, intermediate 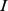 and repressor 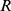. It is interesting to observe that the dynamics of this system is determined by kinetics of the three components represented by each of 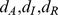 and the cooperativity of the feedback repression 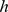.

The Jacobian of this system has the structure:
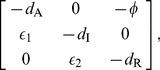
(3)where 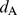,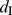,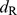,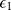,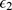 and 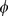 are all positive. 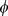 is a measure of the feedback repression, 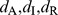 are the effective degradation rates, 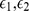 are activation strength along the chain, all evaluated at steady state. Applying the Routh-Hurwitz criterion to the Jacobian in (3) produces a condition for oscillations [6]:
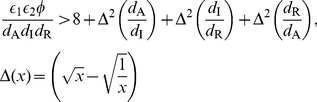
(4)


It is clear that the right hand side of (4) is minimized by having balanced degradation at the critical point, i.e., 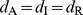. If the right hand side is not balanced, the condition for producing oscillations becomes harder to satisfy.

### Core motif

In the core motif, solving for the critical point turns (4) into
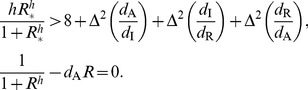
(5)


It is evident from this expression that if degradation rates in the system are balanced, then 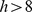 is the condition for oscillations [6]. The minimal 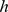 requirement is obtained by a critical point 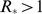 by maximizing 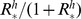, which requires degradation rate 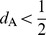.

### Self-activating positive feedback motifs

The three self-activating motifs we consider are of the form:
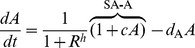


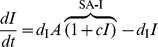


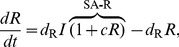
(6)


An inspection of (6) reveals that this system has the same Jacobian structure (3) as the core motif. Then, (4) turns into
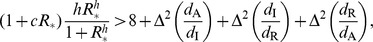


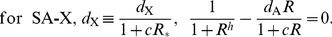
(7)


The condition in (7) is more easily satisfied (i.e., for a smaller *h*) than (4) due to (a) the multiplicative factor on the left hand side greater than 1 (b) an increase in the repressor levels at the critical point 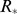, making making 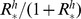 larger for any given *h*. Despite these two favorable factors, the altered effective degradation rate at the critical point might increase the right hand side due to increased mismatch between the degradation rates. It appears from numerical simulations, that the former two factors outweigh the latter and make self-activation always favorable from the viewpoint of reducing cooperativity.

### Michaelis-Menten degradation motifs

These three motifs can be described as:
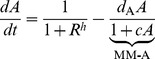


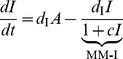


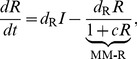
(8)


This system too has the same Jacobian structure (3) and therefore we reach a similar criterion for oscillations:
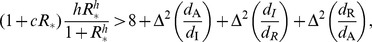


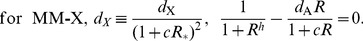
(9)


(9) is easily seen to be exactly the same as the criterion for oscillations in the self-activation motif (7) with only one key difference. With MM degradation the effective degradation rate of the component with the positive feedback is reduced much more than with self-activation. This results in parameter sets with significant increases in the right hand side of (9) due to mismatches in degradation. Thus, in the Monte-Carlo simulations presented in the Results section, the MM motif oscillates with a smaller cooperativity if the MM degradation does not significantly increase the mismatch between the effective degradation rates (thereby increasing the right hand side of (9)).

### Cross-acting feedback motifs

The three CA motifs are implemented as:
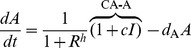


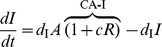


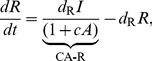
(10)


The CA motifs have a different Jacobian structure than the MM and SA motifs. The Jacobian for CA motifs has an additional non-zero element 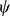 whose position and sign depends on the particular motif:
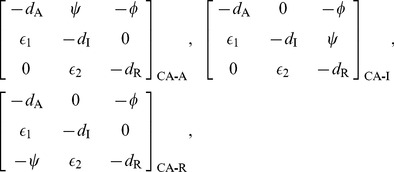
(11)where the strength of the cross-acting feedback 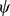 is positive. In all these cases, however, the Routh-Hurwitz criterion for oscillations has the form:
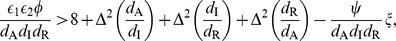


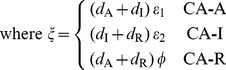
(12)


While the criterion for oscillations with cross-acting feedback (12) is similar to the core motif (4), the added term arising from the feedback reduces the effect of the mismatches in degradation rates of the components. Alternatively, the added term can be viewed as reinforcing the loop gain of the system (represented by the left hand side). Thus, we expect the CA motifs to oscillate with smaller Hill coefficients. Moreover, the reduction of the right hand side by the feedback (12) is maximized when the degradation rates of the components involved in the feedback are the largest (or the half-lives of those components are the least). However, the CA-R motif behaves differently, since the added term is also proportional to feedback repression metric 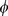. So, with increased feedback repression (larger *h*), the mismatch costs can be reduced significantly more than the CA-A and CA-I motifs, where all parts of the added term are constants.

### Identifying positive and negative feedbacks from the Jacobian

We have shown that positive feedback promotes oscillations in the three-component negative feedback motif. In order to recognize such features in oscillator motifs, a systematic approach to identifying feedback loops is necessary. One common approach, that we adopt here, is to identify feedback loops using the system Jacobian that we computed above.

A feedback loop is the causal effect of a component on itself often mediated by other system components. Given the Jacobian with elements 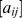, 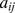 is the effect of a change in component 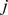 on component 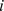, i.e., 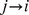. Therefore, the effect of component 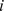 on itself mediated by no other component is 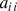, by one other component 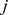 (a two-component feedback loop), i.e., 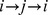, is 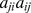, and so on. We refer to the product 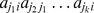 representing a feedback loop consisting of 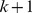 components as the *gain* of the 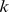-component feedback loop 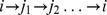. A positive and negative gain represent a positive and negative feedback loop, respectively.

In the core motif with Jacobian in (3), we find one three-component feedback from 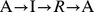 with gain of 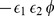, i.e., a negative feedback loop. There are further three one-component negative feedbacks due to the three degradation terms, 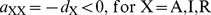.

The SA and MM class of motifs have the same Jacobian structure as the core motif and hence have the same three-component negative feedback loop and three one-component degradation-related feedbacks. However, as shown in (7) and (9), the gain of one of the one-component negative feedback loops is reduced by the positive feedback (compare effective degradation rates between the two equations and (4)). In other words, the positive feedbacks in the MM and SA motifs shown in Figure 1B work concurrently with the one-component negative feedback present in the core motif. On the other hand, the Jacobians of CA class in (11) have, in addition to the feedbacks in the core motif, an additional two-component positive feedback, for e.g., the CA-A motif has a two-component positive feedback loop 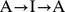 with gain 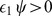.

## Supporting Information

Figure S1 **Distribution of the amount of reduction in cooperativity with positive feedback.** The data in Figure 2 is revisualized (and kernel-smoothed) based on the amount by which positive feedback reduces cooperativity using the same color coding for the different motifs. Note that a positive value on the x-axis represents a reduction in the cooperativity by that amount.
(TIF)

Figure S2
**Relationship between the strength of positive feedback (measured by the parameter** 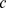 **) and required degree of cooperativity.** As the cooperativity is increased the system starts oscillating after undergoing a Hopf bifurcation. Thus, the Hopf bifurcation lines shown represent the boundary between non-oscillatory and oscillatory regimes. Small cooperativity and weak positive feedback never lead to oscillations. For 50 different random choices of degradation rates of the three components, the boundary between the two regimes for each of the positive feedback motifs in Figure 1B is shown. Notice how the boundary shifts to higher cooperativity at high positive feedback strengths for the MM and CA-R motifs.(TIF)
