# Supplementary figures and images for: Positive Feedback Promotes Oscillations in Negative Feedback Loops
Source: PLoS One. 2014 Aug 15;9(8):e104761. doi: 10.1371/journal.pone.0104761 (PMC4134231; doi:10.1371/journal.pone.0104761)

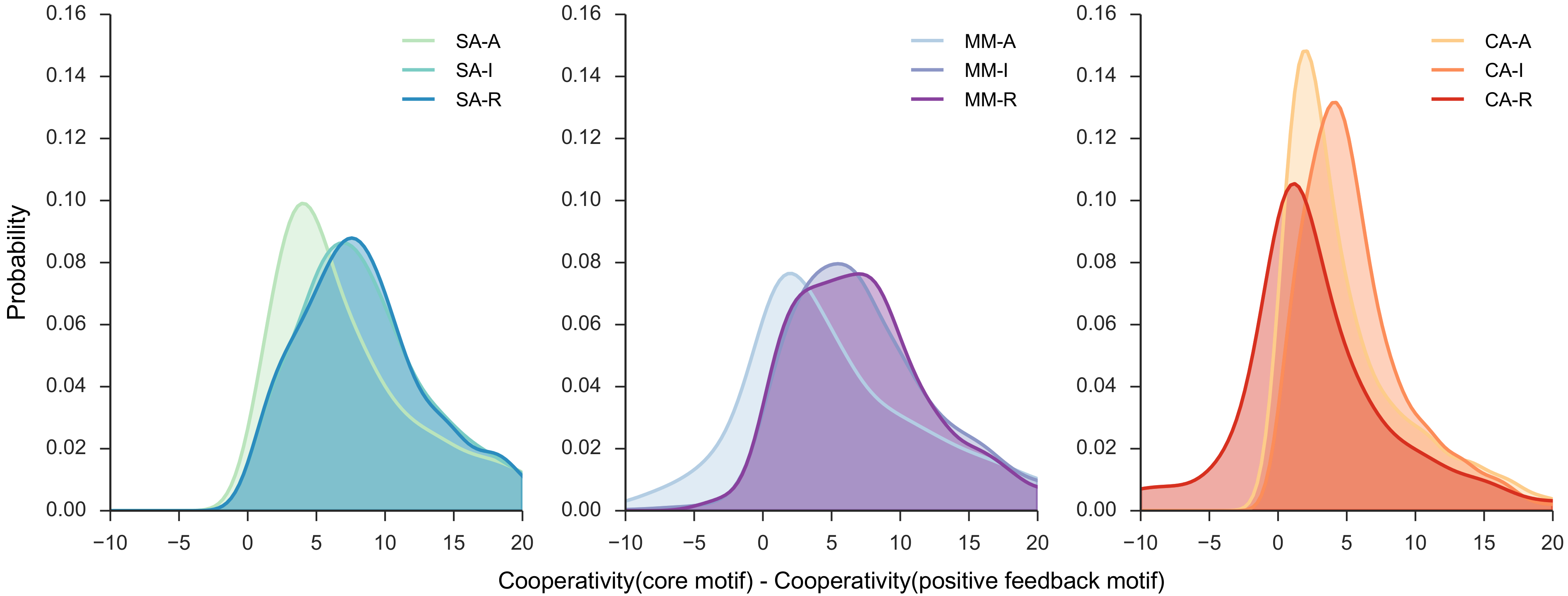

Supplement: Figure S1 — Distribution of the amount of reduction in cooperativity with positive feedback. The data in Figure 2 is revisualized (and kernel-smoothed) based on the amount by which positive feedback reduces cooperativity using the same color coding for the different motifs. Note that a positive value on the x-axis represents a reduction in the cooperativity by that amount. (TIF) [file pone.0104761.s001.tif]

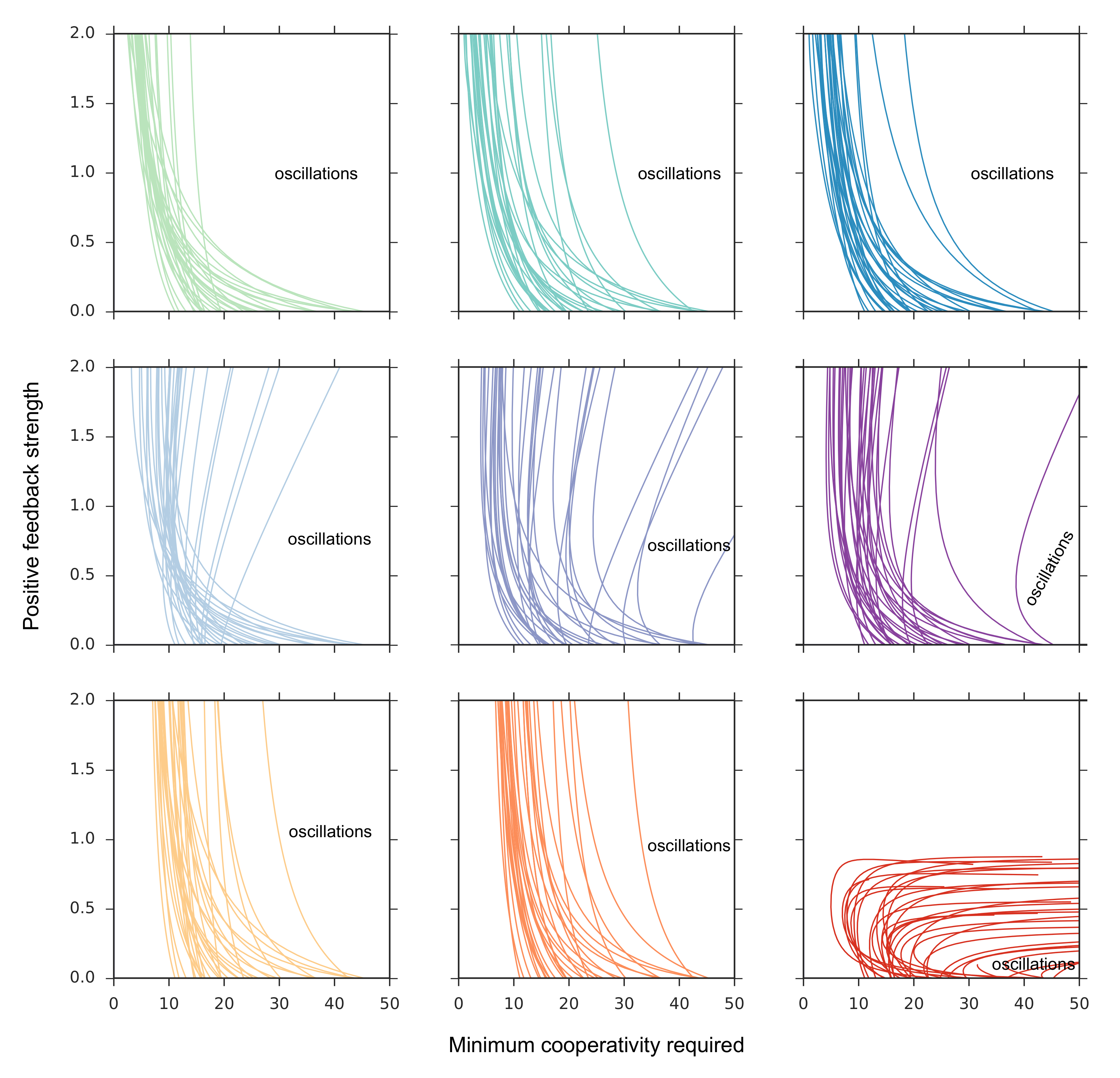

Supplement: Figure S2 — Relationship between the strength of positive feedback (measured by the parameter ) and required degree of cooperativity. As the cooperativity is increased the system starts oscillating after undergoing a Hopf bifurcation. Thus, the Hopf bifurcation lines shown represent the boundary between non-oscillatory and oscillatory regimes. Small cooperativity and weak positive feedback never lead to oscillations. For 50 different random choices of degradation rates of the three components, the boundary between the two regimes for each of the positive feedback motifs in Figure 1B is shown. Notice how the boundary shifts to higher cooperativity at high positive feedback strengths for the MM and CA-R motifs. (TIF) [file pone.0104761.s002.tif]
